# Mineralization Potential of Electrospun PDO-Hydroxyapatite-Fibrinogen Blended Scaffolds

**DOI:** 10.1155/2012/159484

**Published:** 2012-08-16

**Authors:** Isaac A. Rodriguez, Parthasarathy A. Madurantakam, Jennifer M. McCool, Scott A. Sell, Hu Yang, Peter C. Moon, Gary L. Bowlin

**Affiliations:** ^1^Tissue Engineering Laboratory, Department of Biomedical Engineering, School of Engineering, Virginia Commonwealth University, East Hall, Room E1254, 401 W. Main St, P.O. Box 843067, Richmond, VA 23284-3067, USA; ^2^School of Dentistry, Virginia Commonwealth University, Richmond, VA 23298-0566, USA; ^3^Physical Medicine and Rehabilitation Service, Hunter Holmes McGuire VA Medical Center, Richmond, VA 23249, USA; ^4^Biomaterials Laboratory, School of Dentistry, Virginia Commonwealth University, Richmond, VA 23298-0566, USA

## Abstract

The current bone autograft procedure for cleft palate repair presents several disadvantages such as limited availability, additional invasive surgery, and donor site morbidity. The present preliminary study evaluates the mineralization potential of electrospun polydioxanone:nano-hydroxyapatite : fibrinogen (PDO : nHA : Fg) blended scaffolds in different simulated body fluids (SBF). Scaffolds were fabricated by blending PDO : nHA : Fg in the following percent by weight ratios: 100 : 0 : 0, 50 : 25 : 25, 50 : 50 : 0, 50 : 0 : 50, 0 : 0 : 100, and 0 : 50 : 50. Samples were immersed in (conventional (c), revised (r), ionic (i), and modified (m)) SBF for 5 and 14 days to induce mineralization. Scaffolds were characterized before and after mineralization via scanning electron microscopy, Alizarin Red-based assay, and modified burnout test. The addition of Fg resulted in scaffolds with smaller fiber diameters. Fg containing scaffolds also induced sheet-like mineralization while individual fiber mineralization was noticed in its absence. Mineralized electrospun Fg scaffolds without PDO were not mechanically stable after 5 days in SBF, but had superior mineralization capabilities which produced a thick bone-like mineral (BLM) layer throughout the scaffolds. 50 : 50 : 0 scaffolds incubated in either r-SBF for 5 days or c-SBF for 14 days produced scaffolds with high mineral content and individual-mineralized fibers. These mineralized scaffolds were still porous and will be further optimized as an effective bone substitute in future studies.

## 1. Introduction

Observed in approximately 1 in 700 live births, cleft lip (with or without cleft palate) is the most common congenital craniofacial birth defect in humans [1–3]. Cleft palate occurs when the palatal shelves do not fuse properly [4]. Current treatments involve surgeries which rotate adjacent soft tissue into the defect site and secondarily graft hard tissue into the cleft defect [5]. The most common hard tissue graft is an autograft, whereby bone is taken from the patient's own body and reimplanted into the defect site. Autologous bone grafts harvested from patient donor sites are osteoconductive (provide a scaffold where bone cells can proliferate), osteoinductive (induce proliferation of undifferentiated cells and their differentiation into osteoblasts), and osteogenic (provide a reservoir of skeletal stem and progenitor cells that can form new bone) [6]. However, autografts are limited in availability, require additional invasive surgery, and have donor site morbidity; all of which encourage the development of alternative bone substitutes [6–11]. Biomaterials such as ceramics, cements, glasses, metals, polymers, and composites have been studied for their ability regenerate bone [12–16]. Of these, polymers have emerged as the material of choice for scaffolds intended for bone tissue engineering due to their inherent biocompatibility and the ability to tailor physiochemical properties and degradation rates [17]. Thus, we proceeded with fabricating polymer scaffolds via electrospinning to produce extracellular matrix (ECM) mimicking substitutes for bone tissue engineering.

Bone is a natural composite of collagenous organic matrix reinforced by an inorganic mineral phase of hydroxyapatite (HA) whose structure is ultimately responsible for its functional properties. Other components of bone include calcium phosphates, water, proteins, and so forth [18]. Natural bone is a complex and highly organized structure of parallel collagen nanofibrils, and carbonated apatite (HA nanocrystals, (nHA)) located within the fibrils and precipitated on their surface [18, 19]. These HA nanocrystals can be either platelet or spindle shaped and up to about 200 nm long which creates a large surface area for effective mineral exchange [20]. The collagen ECM is the foundation upon which minerals are deposited. Ultimately, the biomechanical properties and biological function is attributed to this organic-inorganic nanocomposite bone ECM [21]. Our lab has developed a method to successfully incorporate nHA within polymeric electrospun nanofibers to mimic the structure of native bone ECM [22]. 

A bone-like mineral (BLM) layer formed on the surface of biomaterials is an essential requirement for the material to bond to the living bone and enhance osteoconductivity. Simulated body fluid (SBF) has been used previously to induce mineral nucleation creating a BLM layer on the surface of materials. SBF has been widely used for biomimetic BLM coating on bioinert materials to directly mimic the process of mineralization in native bone and to predict the *in vivo* bioactivity of the material [11, 22–29]. It has been found that the quicker a material can form a BLM layer in SBF, the better suited it is for quickly bonding to the host bone [30]. 

Natural polymers attract special interest in tissue engineering since they are biocompatible, biodegradable, and natural substrates where cells can attach, proliferate, and function [31]. Due to its excellent bioactivity, biocompatibility, ability to induce cellular interaction, and subsequent scaffold remodeling, fibrinogen (Fg) is one such natural biopolymer of interest for tissue engineering [32–34]. Fg is a highly abundant plasma protein (340 kDa, globular) and consists of six polypeptide chains: 2A*α*, 2B*β*, and 2*γ* [35]. Fg functions as the main structural component in clot formation and wound repair [36, 37]. In addition to its role in clotting, Fg is a protein which has the capacity to bind a variety of molecules [32]. This property is of interest when evaluating the mineralization potential of electrospun Fg scaffolds since it may provide more nucleation sites for mineral deposition. Previous studies have reported on the use of electrospun Fg scaffolds for tissue engineering applications [32–34, 38]. However, the potential of electrospun Fg scaffolds for bone tissue engineering has yet to be explored. Combining the bone-bioactivity of the inorganic materials with the structural integrity of the organic polymers introduces an organic-inorganic composite bone tissue engineering scaffold which can potentially serve as a substitute for autologous grafts for cleft palate repair.

## 2. Materials and Methods

### 2.1. Electrospinning

Electrospun scaffolds were fabricated with varying ratios of polydioxanone (PDO, Ethicon, Inc.), Fg (Fraction 1, Type 1-S from bovine plasma, Sigma-Aldrich, Co.), and HA nanopowder (particle size < 200 nm (BET), Sigma-Aldrich). PDO and Fg were dissolved in 1,1,1,3,3,3 hexafluoro-2-propanol (HFP, TCI America) at 100 mg/mL. nHA was added as a wt% of the polymer and sonicated for 10 minutes on pulse mode (on: 50 s, off: 10 s) at 38% maximum amplitude using a Cole-Palmer Ultrasonic Processor sonicator (model CPX 750). Sonicating was necessary to ensure proper dispersal of the mineral since nHA sedimented in HFP. To this sonicated solution, a known amount of polymer was added to attain the final concentration. It has been shown that by using this method of preparing polymer-nHA solutions, nHA is successfully incorporated within the composite scaffold [22]. Different scaffold types were fabricated by blending PDO : nHA : Fg in the wt% following ratios: 100 : 0 : 0, 50 : 25 : 25, 50 : 50 : 0, 50 : 0 : 50, 0 : 0 : 100, and 0 : 50 : 50. Fg solutions were made with a 90% by volume solution of HFP and 10% by volume of 10x minimal essential medium (MEM, Sigma Aldrich, Co.). Solutions were left for 24 hours on a shaker plate to ensure that all of the Fg dissolved to form a homogeneous solution [39]. In order to achieve PDO-Fg blended scaffolds, a calculated volume of Fg solution was transferred to a known volume of PDO solution, briefly vortexed, and placed on a shaker for 5 minutes prior to electrospinning. All solutions were loaded into a 5 mL syringe (Luer-Lok Tip, Becton, Dickenson, and Company) and placed in a KD Scientific syringe pump (model 100) to dispense the solutions at a constant rate. A high-voltage power supply (Spellman CZE1000R, Spellman High Voltage Electronics Corporation) was used to apply a voltage to a blunt needle tip. Electrospinning parameters were optimized for each solution (Table 1) in order to generate continuous nonwoven composite nanofibers. Randomly oriented fibers were collected onto a rotating, grounded, flat, stainless steel rectangular mandrel (7.5 × 2.5 × 0.5 cm). Scaffolds were removed from the mandrel after electrospinning and dried in the hood for 30 minutes. Using a dermal biopsy punch (AcuPunch, Acuderm inc.), 10 mm discs were punched and used for all biomimetic mineralization experiments.

### 2.2. Biomimetic Mineralization

The ionic concentrations of the commonly used simulated body fluid, conventional SBF (c-SBF), are not exactly equal to those of blood plasma. Oyane et al. made revisions to c-SBF and created three new SBFs (revised (r), ionic (i), and modified (m)) that have ionic concentrations equal to, or closer to, those of blood plasma (Table 2). The ionic concentrations of the r-, i-, and m-SBFs were formulated to equal those of total blood plasma, dissociated blood plasma, and total blood plasma (except for HCO_3_^−^), respectively. For this reason we prepared different types of 1x SBFs by following the protocol published by Oyane et al. [40]. Eight 10 mm (diameter) discs were punched from each of the electrospun scaffold compositions and separately incubated in 2 mL of 1x c-, r-, i-, or m-SBF for 5 and 14 days at 37°C and 5% CO_2_ atmosphere. SBF solutions were freshly prepared and replenished every 5 days. These experiments were performed under static conditions in standard tissue culture grade 24-well plates (Costar, Corning Incorporated).

At the end of the experiment, scaffolds were removed and rinsed with DI water to wash off any minerals that were not bound to the scaffold. To visually inspect surface mineralization, one scaffold disc was dehydrated and used for scanning electron micrographs (SEM). For mineral quantification, three scaffolds were used for alizarin red S (ARS) staining and the remaining four scaffolds were analyzed using the burnout test to calculate the percent mineral composition of the scaffolds. 

### 2.3. Scaffold Characterization

SEM was performed in order to evaluate the scaffold and fiber surface characteristics prior to and following mineralization. Samples of electrospun scaffolds for SEM were dehydrated, mounted on aluminum stubs, sputter coated in gold for 90 seconds (Electron Microscope Sciences model 550), and examined using a Zeiss EVO 50 XVP scanning electron microscope. Fiber diameters were calculated from the SEM images using UTHSCSA Image Tool 3.0 software.

### 2.4. Alizarin Red S Staining

ARS is a dye that selectively binds to calcium salts. ARS staining was used to quantify mineralization by modifying a published protocol [41]. Scaffolds were fixed in 1 mL of formaldehyde for 10 minutes then stained with 1 mL of 40 mM alizarin red (pH adjusted to 4.1) for 30 minutes. All scaffolds were rinsed repeatedly in the well plates with DI water until all unbound dye was washed off (wash solution became clear and lost its red/pink tint). The samples were transferred to 2 mL tubes containing 1.5 mL of 50% acetic acid. Scaffolds were left in acetic acid for 18 hrs at room temperature to ensure that all of the bound dye was dissolved. After the 18 hr destain, 500 *μ*L of the solubilized stain was pipetted into a 1.5 mL tube containing 600 *μ*L of 1 M NaOH in order to adjust the pH to 4.1. 200 *μ*L of this solution was then transferred to a 96-well plate and absorbance measured at 550 nm using a SpectraMax Plus 384 Microplate Spectrophotometer (Molecular Devices).

### 2.5. Burnout Test

The mineral content in the scaffolds was quantified by modifying a published burnout test protocol [42]. Duration of burning was optimized for each scaffold type to ensure that all of the organic components were burned off leaving only the electrospun nHA and newly deposited minerals. 

To determine burning times, the original nonmineralized scaffolds were burned at 500°C in a platinum crucible (Engelhard-Clal, item 201-20CC) until the scaffolds were completely disintegrated. Original electrospun scaffolds that did not contain nHA were burned until nothing remained in the crucible. Scaffolds containing nHA were burned until only the dry inorganic nHA powder remained in the crucible. By weighing the remaining nHA powder in the crucible after burning we were able to mathematically determine the efficiency of incorporating HA into electrospun scaffolds as well as determine how much HA is lost during the electrospinning process. The required burn times for each scaffold type were recorded (Table 3) and applied to the same scaffold composition after mineralization. 

After mineralizing for 5 or 14 days, scaffolds were rinsed, air dried for 24 hours, and then placed in a crucible of a known weight. Scaffolds were weighed (*W*_1_), then inserted into a muffle furnace (KH Huppert Co., Type ST, Style 2A) at 500°C for the desired time. A voltage rheostat (The Superior Electric Co., Powerstat, Type 116) was used as a voltage divider to keep the furnace at 500°C throughout burning. Remaining mineral in crucible after burning was weighed (*W*_2_) and (*W*_2_/*W*_1_)∗100 was used to determine the percent mineral composition of the 3D scaffolds before and after mineralization. All weights were measured using a Christian Becker Analytical Balance scale (Style AB-4) accurate to 0.0001 grams.

Control experiments were conducted in order to verify that the HA was not burned off though the burnout test. For the first control experiment, 0.2 grams of the original powdered nHA was placed in the crucible and burned for three hours. In the second experiment 0.25 grams of powder nHA was sonicated in 5 mL of HFP. After sonication the solution was left uncapped under a fume hood for 24 hours for the HFP to evaporate leaving only the dry sonicated nHA. This nHA was scraped off into the crucible and also burned for three hours. A burning duration of three hours was chosen because this was the maximum burn time that the scaffolds were be subjected to. After three hours in the muffle furnace, no loss or gain in weight to either of the HA samples should be observed. This confirms that any inorganic components left in the crucible after burning the original scaffolds are attributed to the incorporated electrospun nHA. 

### 2.6. Statistical Analysis

Statistical analysis was performed using JMP in 4 statistical software (SAS Institute) to determine significant differences between fiber diameters as well as ARS absorbance values. Analysis of the data was based on a Kruskal-Wallis one-way analysis of variance on ranks and a Tukey-Kramer pairwise multiple comparison procedure. The results are presented in mean ± SD.

## 3. Results 

### 3.1. Scanning Electron Microscopy

**Figure 1:**
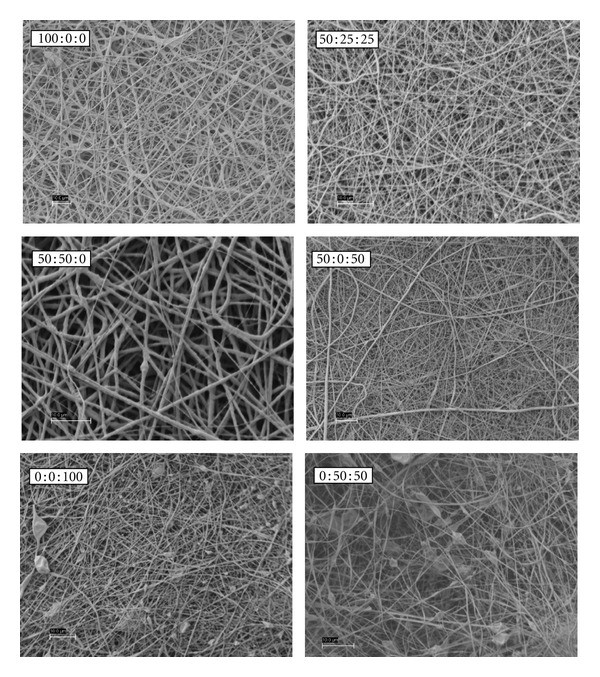
Original electrospun PDO : nHA : Fg scaffolds.

**Figure 2:**
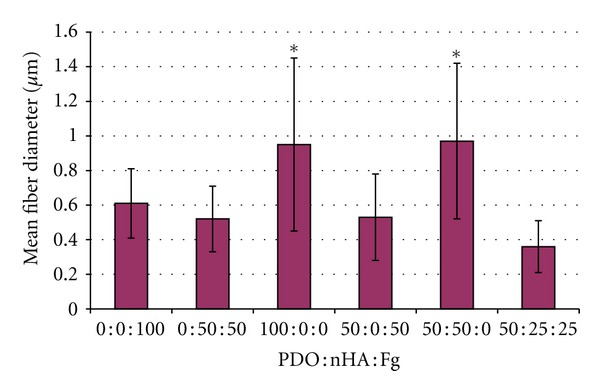
Fiber diameters of PDO : nHA : Fg scaffolds. *P* < 0.0001.

SEM images of PDO : nHA : Fg original electrospun scaffolds are illustrated in Figure 1. All scaffolds contained continuous, randomly oriented fibers with the following fiber diameters: 100 : 0 : 0 (0.95 ± 0.5 *μ*m), 50 : 50 : 0 (0.97 ± 0.45 *μ*m), 50 : 25 : 25 (0.36 ± 0.15 *μ*m), 50 : 0 : 50 (0.53 ± 0.25 *μ*m), 0 : 0 : 100 (0.61 ± 0.2 *μ*m), and 0 : 50 : 50 (0.52 ± 0.19 *μ*m). Scaffolds without Fg (100 : 0 : 0 and 50 : 50 : 0) had larger fibers when compared to scaffolds containing Fg (Figure 2, *P* < 0.0001). 

**Figure 3:**
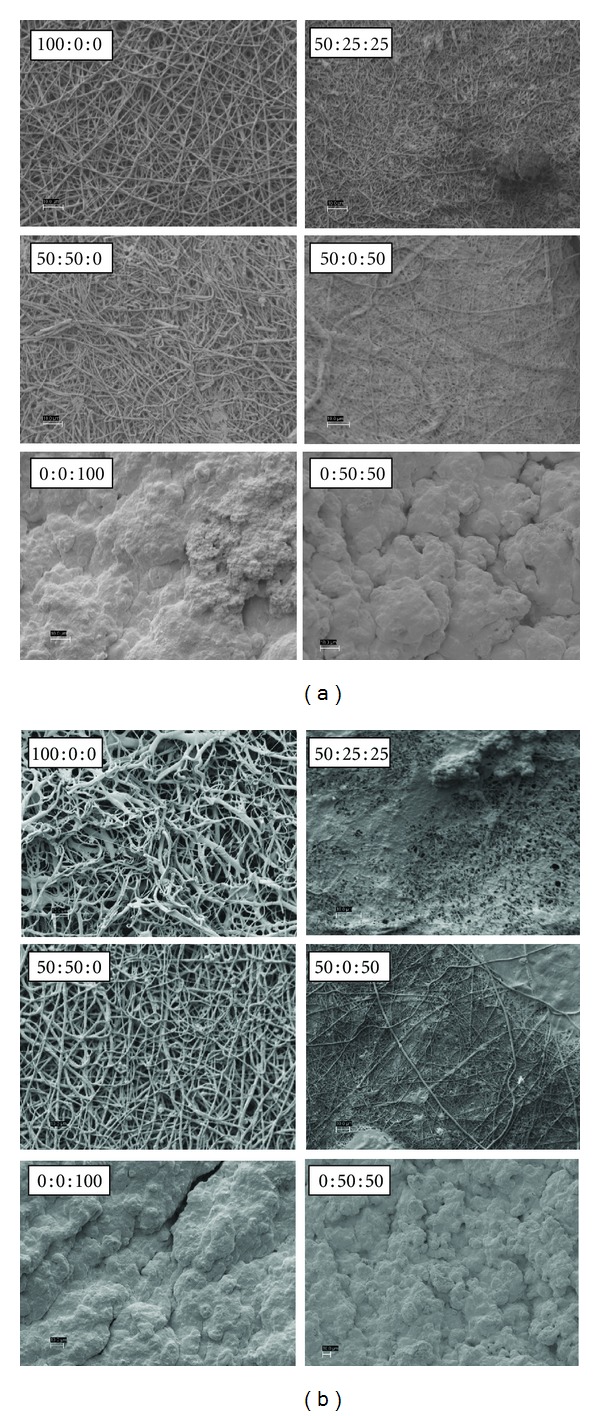
PDO : nHA : Fg Scaffolds Incubated in c-SBF for 5 days (left 2 columns) and 14 days (right 2 columns), scale bar = 10 *μ*m.

**Figure 4:**
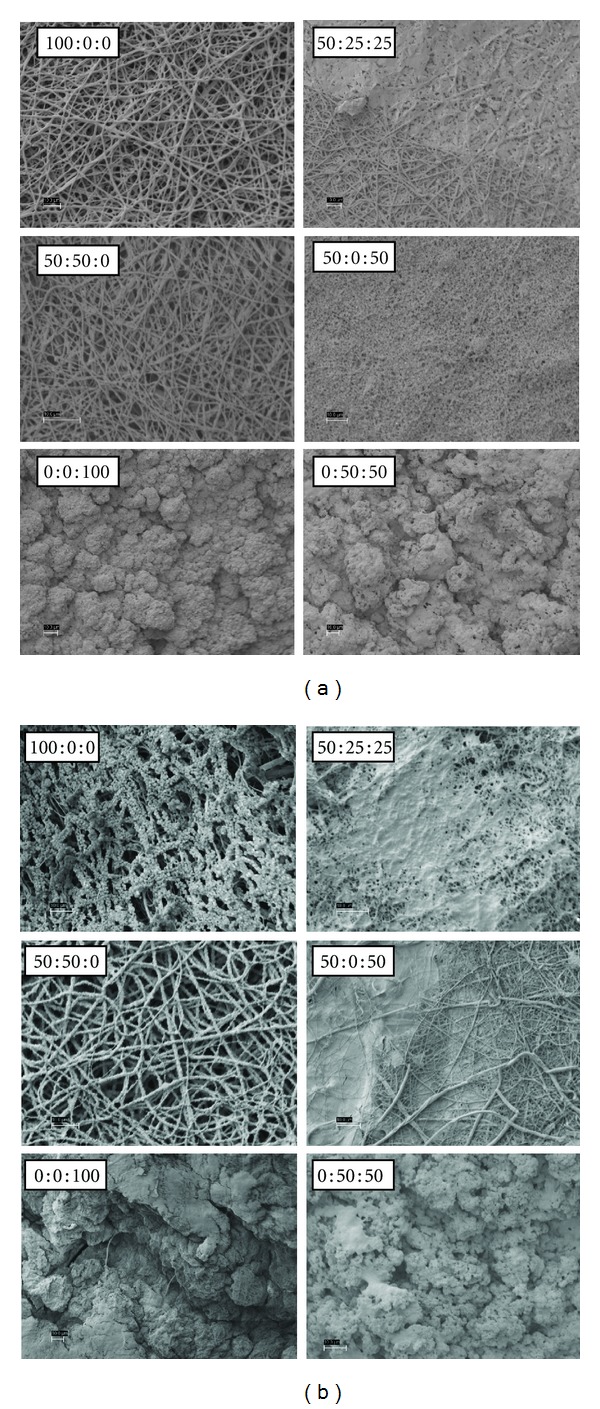
PDO : nHA : Fg scaffolds incubated in r-SBF for 5 days (left 2 columns) and 14 days (right 2 columns), scale bar = 10 *μ*m.

**Figure 5:**
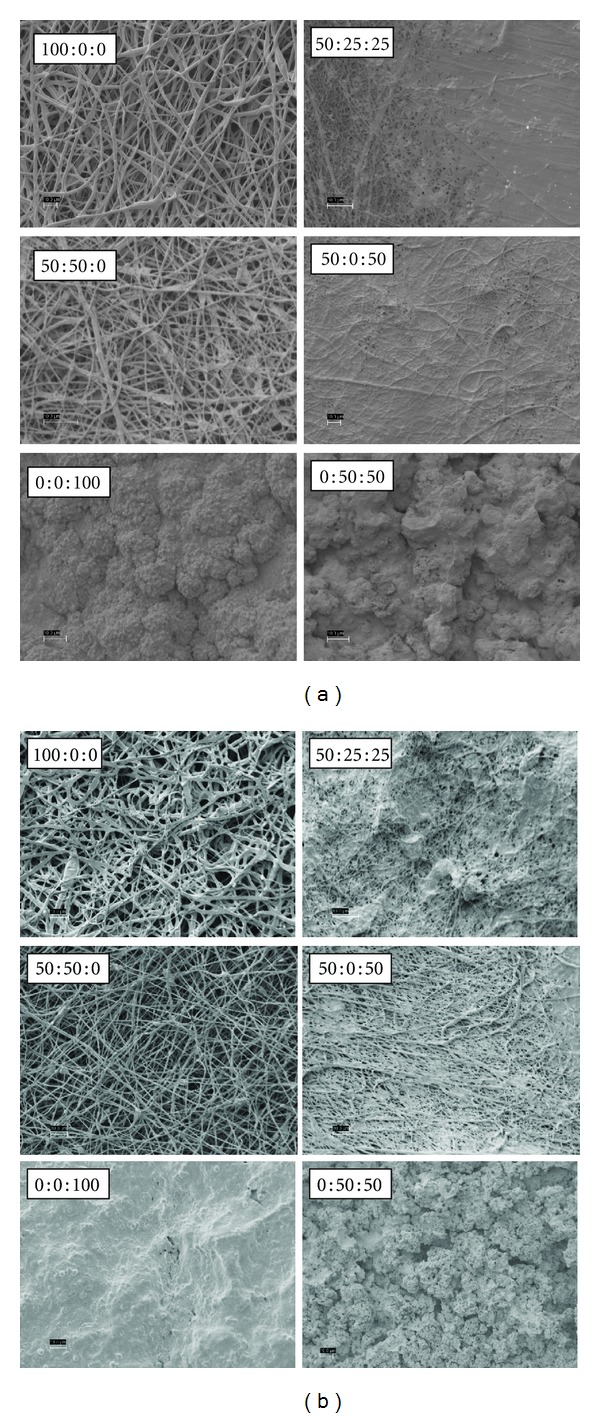
PDO : nHA : Fg scaffolds incubated in i-SBF for 5 days (left 2 columns) and 14 days (right 2 columns), scale bar = 10 *μ*m.

**Figure 6:**
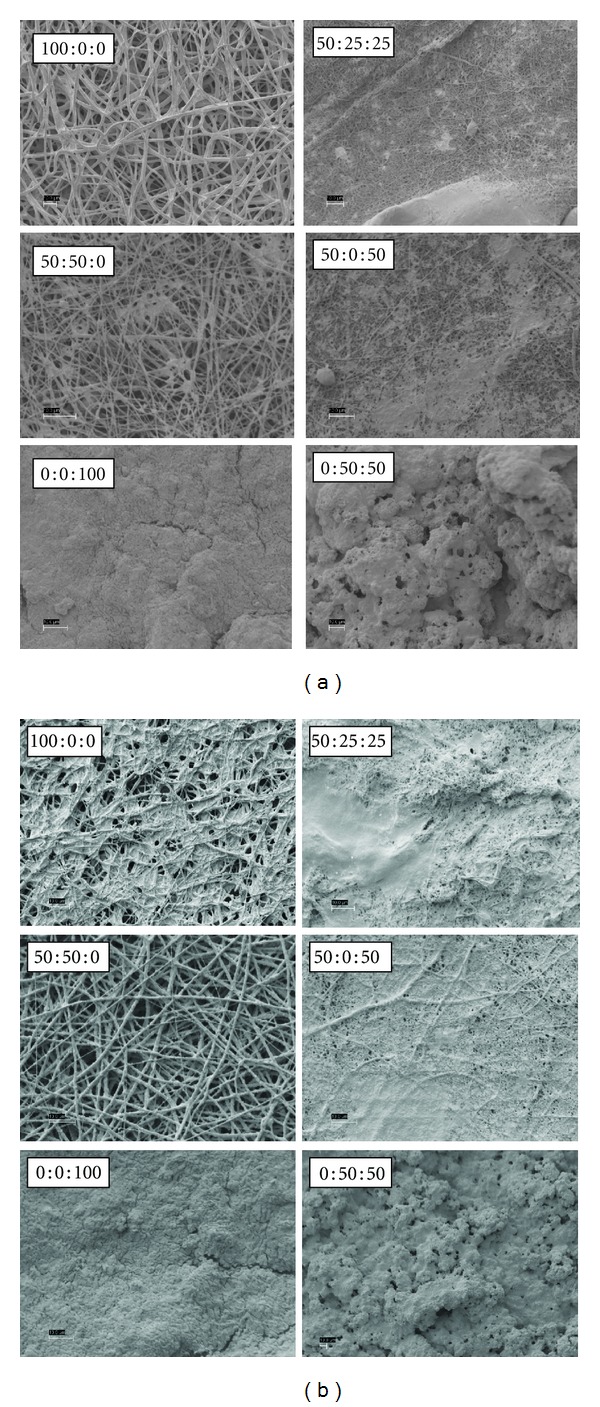
PDO : nHA : Fg scaffolds incubated in m-SBF for 5 days (left 2 columns) and 14 days (right 2 columns), scale bar = 10 *μ*m.

Figures 3, 4, 5, and 6 are SEM images of all PDO : nHA : Fg scaffold blends incubated in 1x, c-, r-, i-, and m-SBF for both 5 and 14 days to induce mineralization (scale bars at 10 *μ*m). With the exception of a pure PDO scaffold (100 : 0 : 0), the pattern of mineral deposition induced by each scaffold type was consistent throughout SBFs. A scaffold composition of 100 : 0 : 0 incubated for 14 days in various SBFs exhibited different apatite formation. When immersed in c-SBF, 100 : 0 : 0 scaffolds began to degrade and lose their continuous nanofibrous structure. In r-SBF, mineralization occurred on individual fibers in a bead-like formation. In i-SBF, a thin layer of minerals began to grow on the outer layer of fibers, however, this effect was even more prominent in m-SBF.

### 3.2. Alizarin Red S Staining

Fg scaffolds without PDO (0 : 0 : 100 and 0 : 50 : 50) were not analyzed for mineral content via ARS because these scaffolds lost mechanical integrity after 5 days incubation in SBFs and could not be salvaged for staining. As expected, original electrospun scaffolds containing nHA had higher ARS absorbance values than mineralized scaffolds: 100 : 0 : 0 (0.0426 ± 0.0004), 50 : 0 : 50 (0.0547 ± 0.0012), 50 : 25 : 25 (0.1889 ± 0.0122), and 50 : 50 : 0 (0.2304 ± 0.0397). When scaffolds are mineralized it is possible for different apatites to form (carbonated apatite, Ca-P, HA, etc.). It is important to note that the original nonmineralized scaffolds either contained no nHA or pure nHA. ARS may have a different binding affinity to these various apatite structures. The environment in which the scaffolds were incubated is also an important consideration, specifically the 5% CO_2_. An increase in the CO_2_ concentration of the SBF will lower the pH of the mineralizing solution. HA becomes more soluble at a lower pH [43] suggesting that HA may go into solution ultimately demineralizing the scaffold resulting in a lower absorbance value. The binding affinity of ARS to various apatites and the solubility of HA are both factors to consider when comparing the lower absorbance values of mineralized scaffolds to the original scaffolds. Therefore, no statistics were performed to compare the original and days 5 and 14 mineralized scaffolds. 

It is evident from SEM images that each scaffold nucleates apatites differently. Scaffolds without nHA most likely attract Ca-P apatites during primary nucleation since these are the beginning steps to forming bone. Scaffolds containing nHA may attract different apatites to their surface since nHA is a higher form of Ca-P. Considering the above possibilities, statistics were performed to determine differences of SBF treatment on each scaffold type. 

**Figure 7:**
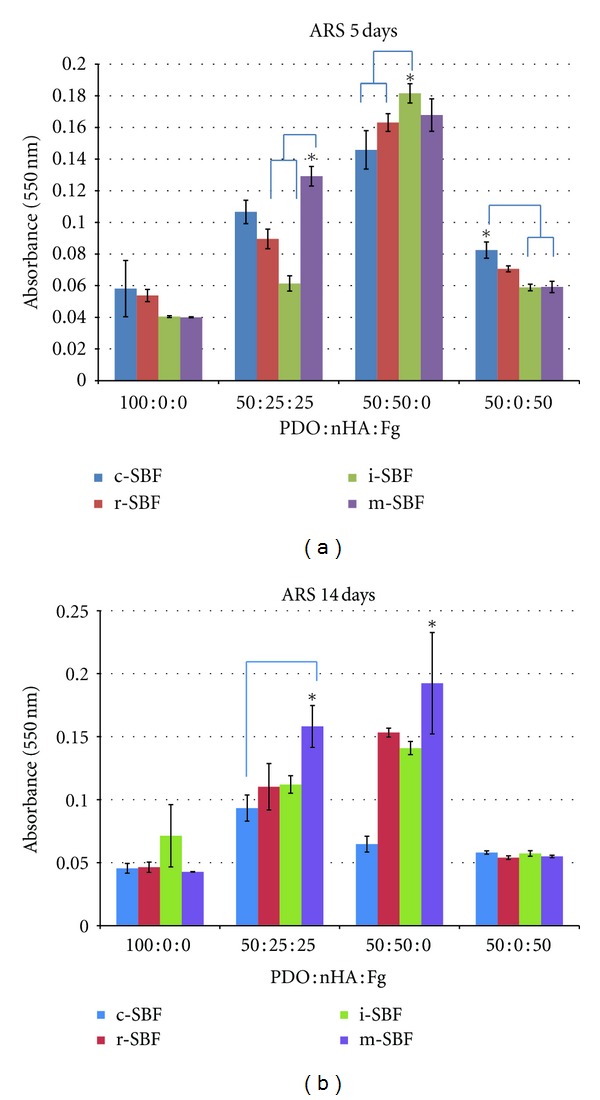
ARS data and statistics for mineralized scaffolds. *Denotes statistical significance (*P* < 0.05).

The absorbance values of the mineralized scaffolds and the effect of different SBFs on each scaffold composition is displayed in Figure 7. The control value for a pure PDO scaffold was 0.0426. After 5 days incubation, the absorbance values of 100 : 0 : 0 scaffolds in all SBFs were not significantly different (*P* < 0.05) from each other. An average-low value of 0.0481 suggests that 100 : 0 : 0 scaffolds induced little to no mineralization independent of SBF type. Incubating 50 : 25 : 25 scaffolds in m-SBF resulted in the second-highest absorbance value (0.129) and was significantly different (*P* < 0.05) from r- and i-SBF. This suggests that m-SBF induced more mineralization than r- or i-SBF but was not statistically different (*P* < 0.05) from c-SBF. Immersing 50 : 50 : 0 scaffolds in i-SBF showed the highest absorbance reading (0.182) and was significantly different (*P* < 0.05) from c- and r-SBF. This suggests that i-SBF is superior to c- and r-SBF in mineralization potential for these scaffolds but not statistically significant (*P* < 0.05) from m-SBF. The highest absorbance value for 50 : 0 : 50 scaffolds was observed in c-SBF (0.083). c-SBF was significantly different (*P* < 0.05) from i and m-SBF but not from r-SBF. This implies that c-SBF mineralized 50 : 0 : 50 scaffolds better than i- and m-SBFs, but not r-SBF.

The same statistical analysis of ARS data was performed on scaffolds incubated in SBF for 14 days. Absorbance readings for 100 : 0 : 0 scaffolds in different SBFs were not significantly different from each other. An average-low value of 0.051 suggests that even after 14 days 100 : 0 : 0 scaffolds induced minimal mineralization. For 50 : 25 : 25 scaffolds m-SBF had the highest value (0.158) and was only significantly different (*P* < 0.05) from c-SBF. This implies that m-SBF induced more mineralization than c-SBF but was not statistically different (*P* < 0.05) from r- and i-SBFs. Incubating 50 : 50 : 0 scaffolds in m-SBF resulted in the highest absorbance value (0.193) which was significantly different (*P* < 0.05) from all SBFs. There was no difference between SBFs for 50 : 0 : 50 scaffolds. With an average value of 0.056, little to no mineralization occurred.

The same statistical analysis was used to compare scaffolds compositions for 5 and 14 days incubation in SBF. 100 : 0 : 0 scaffolds showed statistical difference between day 5 and 14. For 50 : 25 : 25 scaffolds, 5 days in m-SBF was only significantly different (*P* < 0.05) than 5 days in i-SBF while 14 days in m-SBF was significantly different (*P* < 0.05) than 5 days in r- and i-SBF and 14 days in c-SBF. This data does not concretely prove that one SBF performs better than the other when comparing day 5 and 14 in 50 : 25 : 25 scaffolds. Statistical analysis performed on 50 : 50 : 0 scaffolds resulted in the same conclusion. However, 50 : 0 : 50 scaffolds incubated in c-SBF for 5 days was statistically significant (*P* < 0.05) to all of the SBFs except i-SBF for 5 days. This suggests that 50 : 0 : 50 scaffolds in either c- or i-SBF for 5 days induce more mineralization than scaffolds incubated in any other SBF for 5 or 14 days.

For both 5 and 14 days, higher absorbance readings were recorded for 50 : 50 : 0 scaffolds showing a visible pattern of increased absorbance values with increased nHA composition. The ARS data suggests that 5 days incubation of 50 : 50 : 0 in SBFs is sufficient to induce mineralization while the other scaffold compositions showed no noticeable difference between 5 and 14 days.

### 3.3. Burnout Test

As previously mentioned, Fg (0 : 0 : 100) and Fg-nHA (0 : 50 : 50) scaffolds degraded after 5 days incubation in SBFs and were not salvaged for mineral quantification via burnout test. The remaining scaffolds were intact and analyzed. 

**Figure 8:**
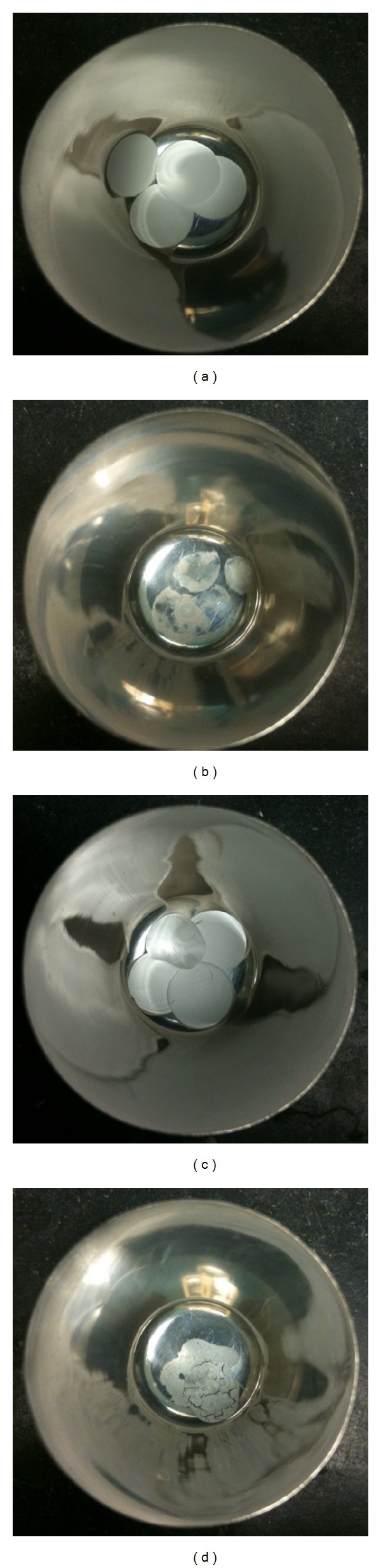
Mineralized scaffolds before and after burnout test. 100 : 0 : 0 scaffolds incubated for 5 days in m-SBF (a) before and (b) after burn. 50 : 50 : 0 scaffolds incubated for 5 days in i-SBF (c) before and (d) after burnout test.

Figure 8 shows images of scaffolds in the crucible before and after burning to provide a macroscopic view of the leftover mineral contents. As predicted, scaffolds that contained nHA had more visible leftover mineral in the scaffold after burning. 

**Figure 9:**
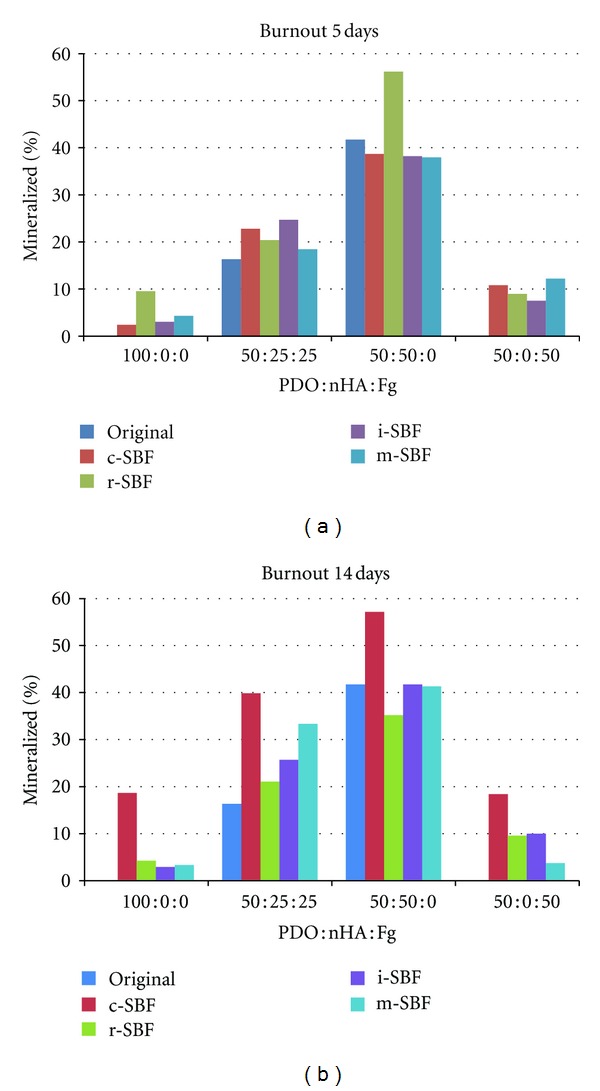
Percent mineral composition of original and mineralized PDO : nHA : Fg scaffolds in different SBFs for 5 and 14 days.

Percent mineral composition of the scaffolds was calculated by weighing scaffolds before and after burning. All PDO : nHA : Fg scaffold compositions were analyzed for mineral content before and after incubation in different SBFs for 5 and 14 days under static conditions (Figure 9). In order to obtain a measurable amount mineral ash, scaffold discs (which were made for triplicates) were combined and burned as one unit. Therefore, no statistics were performed on the preliminary burnout test data; however, observations can still be extracted.

The percent mineral composition of the original electrospun scaffolds prior to mineralization served as the control. The burnout test also proved as a method to measure the effectiveness of incorporating nHA within electrospun scaffolds by determining how much nHA was actually incorporated versus how much was lost during the electrospinning process. Polymer solutions that were prepared without nHA (100 : 0 : 0 and 50 : 0 : 50) yielded a value of 0% mineral. This control verifies that all the PDO and Fg fibers are completely burned. Solutions containing 25% (50 : 25 : 25) and 50% nHA (50 : 50 : 0) yielded values of 16.3% and 41.7%, respectively. This suggests that 34.8% and 16.6% nHA was lost, respectively, during the electrospinning process.

After 5 days incubation in different SBFs all PDO (100 : 0 : 0) scaffolds mineralized to some degree compared to original scaffold mineral composition of 0%. PDO scaffolds incubated in c-, i-, and m-SBFs each were comprised of approximately 3% mineral. Scaffolds incubated in r-SBF had a mineral composition of 9.6%. All 50 : 25 : 25 scaffolds increased in mineral content when compared to the original mineral composition (16%). Scaffolds in c-, r-, i-, and m-SBF contained 22.8%, 20.4%, 24.7%, and 18.4%, respectively. 50 : 50 : 0 scaffolds incubated in c-, i-, and m-SBFs resulted in a slightly lower mineral composition (38%) when compared to the original (41.7%). The highest percent mineral content was observed in 50 : 50 : 0 scaffolds in r-SBF (56.2%). 50 : 0 : 50 scaffolds incubated in all SBFs increased in mineral content from 0% to about 10%. 

Overall the largest percent increase after 5 days was noticed in 50 : 50 : 0 scaffolds in r-SBF. These scaffolds in r-SBF also showed the highest percent mineral composition. By comparing scaffold compositions, it is evident that 50 : 50 : 0 scaffolds contained the highest mineral content before and after mineralization. This was expected since more nHA was incorporated within these scaffolds during the electrospinning process. 

For each scaffold composition, incubation in c-SBF for 14 days resulted in the highest percent mineral content and highest mineral increase due to SBF treatment, suggesting that c-SBF induces the most mineralization independent of scaffold composition. After 14 days, 100 : 0 : 0 scaffolds in c-SBF increased in mineral content from 0% to 18.6% while all other SBFs only resulted in an increase from 0% to 3%. All mineralized 50 : 25 : 25 scaffolds increased in mineral content from 16.3% to 39.8% (c-SBF), 21.1% (r-SBF), 25.7% (i-SBF), and 33.3% (m-SBF). 50 : 50 : 0 scaffolds incubated in i- and m-SBF had no effect on mineral content (41%). Incubation in c- and i-SBF resulted in 57.1% and 29.1% mineral composition, respectively. The difference between c- and i-SBF suggest that 50 : 50 : 0 scaffolds mineralize differently depending on the SBF composition. After 14 days, 50 : 0 : 50 scaffolds increased in mineral content from 0% to various values depending on SBF type. 50 : 0 : 50 scaffolds incubated in c-, r-, i-, and m-SBF resulted in 18.4%, 9.6%, 10%, and 3.7% mineral content, respectively. The difference between c- and m-SBF suggest that 50 : 0 : 50 scaffolds mineralize differently depending on the SBF type. Once again 50 : 50 : 0 scaffolds contained higher percentages of mineral composition before and after mineralization since nHA was originally incorporated within the scaffolds. However, the highest overall mineral increase due to SBF treatment was in 50 : 25 : 25 scaffolds incubated in c-SBF for 14 days. Percent mineral content increased from 16.3% to 39.8% suggesting that 50 : 25 : 25 scaffolds in c-SBF for 14 days induce the most mineralization. 

## 4. Discussion

Kim et al. showed that formed apatites can be different in structure and composition depending on the ionic concentration of SBF used in their formation [44]. They also demonstrated that apatites resembling native bone can be produced if the SBF could be tailored to have ionic concentrations equal to, or close to, those of blood plasma [45]. The SEM images (Figures 3–6) show different patterns of mineralization between SBF solution and scaffold composition. Mineralization patterns varied from beads, individual fiber mineralization, thin sheets of mineral, and dense completely mineralized scaffolds. In the literature, it is debated as to which mineralization pattern is preferred. Most studies have focused on mineralizing scaffolds while maintaining porosity rather than creating a dense BLM layer on the surface of the scaffold. It is thought that the best environment for increasing osteoconductivity, increasing modulus, and enhancing resistance to cellular contractile forces during tissue development exists in a mineralized porous scaffold [8, 23, 24, 28]. For the purpose of this study, the authors were interested in creating mineralized scaffolds with some degree of porosity which will allow cells to recognize the BLM surface while providing space for migration into the scaffold. However, the rapid and high degree of mineralization exhibited by electrospun Fg scaffolds (0 : 0 : 100 and 0 : 50 : 50) was also of interest. 

Overall visual progression of mineralization is observed when increasing incubation period from 5 to 14 days. PDO scaffolds without Fg (100 : 0 : 0 and 50 : 50 : 0) generally mineralized along individual fibers. 100 : 0 : 0 scaffolds have nucleation sites that are more distant from each other since it is a purely synthetic polymer. Depending on the SBF, this could cause continuous mineral growth at sparse sites where calcium phosphate CaP grows on itself forming a bead-like structure at each site. Scaffolds containing nHA (50 : 50 : 0) have more nucleation sites and beads of mineral do not form. HA containing scaffolds induce a more uniform dense individual fiber mineralization. PDO and Fg scaffolds (50 : 25 : 25 and 50 : 0 : 50) showed thin sheet-like mineral deposition throughout. Fg scaffolds not containing PDO (0 : 0 : 100 and 0 : 50 : 50) were entirely mineralized even after 5 days suggesting 5 days immersion in 1x SBF is sufficient to grow a thick BLM layer on the scaffolds. Since Fg has the capability to bind to a variety of molecules, we would expect to see this high degree of mineralization with Fg containing scaffolds.

The addition of Fg did not have an effect on the ARS results when analyzing mineralized scaffolds. Considering the concerns regarding different apatite formation and pH effects, ARS may not be the most accurate method of quantifying mineralization when Fg is incorporated in the scaffold which lead to the preliminary burnout test for mineral quantification. This burnout method will be independent of composition and will be the most accurate for measuring mineralization as long as the sample size is appropriate. If Fg is not present, the ARS method is a more streamlined assay for determining mineral content of the electrospun scaffolds.

From the preliminary burnout test data, some values increased while some decreased from 5 to 14 days incubation in SBF. The increases of mineral content can be attributed to the deposition of minerals on the electrospun scaffolds. The decreases may result from the solubility of nHA at a lower pH as previously described. Scaffolds without nHA (100 : 0 : 0 and 50 : 0 : 50) never surpassed 20% mineral content while 50 : 25 : 25 and 50 : 50 : 0 scaffolds reached up to 40% and 57%, respectively. 50 : 50 : 0 scaffolds in r-SBF for 5 days and 50 : 50 : 0 scaffolds in c-SBF for 14 days contained similar percent mineral compositions (~57%). Since 50 : 50 : 0 scaffolds in r-SBF for 5 days reached a high mineral content just after 5 days, we can assume that these scaffolds will bond to the host bone faster than the same scaffolds in c-SBF for 14 days.

ARS and burnout test data did not support each regarding which SBF type has the highest potential to induce mineralization. There are many variables to consider when quantifying mineralization via ARS which make it difficult to compare the different scaffold compositions. The burnout test is a more reliable method to quantify the mineral content of scaffolds. 

## 5. Conclusion

This study has demonstrated an easy, cost effective approach using synthetic polymers, natural polymers, and inorganic apatites to produce a nanofibrous scaffold for bone tissue engineering applications. By combining the electrospinning of natural and synthetic materials (PDO, Fg, and nHA) and mineralization (SBF) methods, scaffolds with mineralized nanofibers were produced. Also, a reliable method for quantifying mineral content of 3D porous scaffolds before and after mineralization was developed. The degree and type of mineralization was dependent on the scaffold composition, type of SBF, and duration of incubation.

The addition of Fg resulted in smaller fiber scaffolds with thin sheet-like deposition of minerals unlike individual fiber mineralization seen in PDO and PDO-nHA scaffolds. Mineralized electrospun Fg scaffolds without PDO were not mechanically stable after 5 days, but had superior mineralization capabilities which produced a thick BLM layer throughout the scaffolds. Mineral quantification revealed that overall, 50 : 50 : 0 scaffolds had the highest mineral content. 

This preliminary study focused on developing a mineralized porous nanofibrous scaffold intended for cleft palate repair. Results show that Fg containing scaffolds have superior mineralization potential but tend to mineralize as sheets decreasing the porosity of the scaffold. 50 : 50 : 0 scaffolds incubated in either r-SBF for 5 days or c-SBF for 14 days produce scaffolds with high mineral content and individual-mineralized fibers. These mineralized scaffolds were still porous and can potentially serve as effective substrates to induce three-dimensional bone formation. Most importantly, this study demonstrated the high mineralization potential of electrospun Fg scaffolds, which in future work will be studied in more detail to further characterize its mineralization capabilities (e.g., FTIR and X-ray diffraction) within shorter time periods. 

## Figures and Tables

**Table 1:** Electrospinning parameters.

Composition (PDO : nHA : Fg)	Dispense rate (mL/hr)	Air gap distance (cm)	Voltage (kV)	Needle gauge
100 : 0 : 0	3.3	20	26	16
50 : 25 : 25	2	15	29	18
50 : 50 : 0	3.3	20	26	16
50 : 0 : 50	2	13	29	18
0 : 0 : 100	2	11	30	18
0 : 50 : 50	2	11	30	18

**Table 2:** Ionic concentrations of human blood plasma. Modified from [40].

Ion	Concentration (mM)				
	Total blood plasma	c-SBF	r-SBF	i-SBF	m-SBF
Na^+^	142.0	142.0	142.0	142.0	142.0
Cl^−^	103.0	147.8	103.0	103.0	103.0
HCO_3_^−^	27.0	4.2	27.0	27.0	10.0
K^+^	5.0	5.0	5.0	5.0	5.0
Ca^2+^	2.5	2.5	2.5	1.6	1.5
Mg^2+^	1.5	1.5	1.5	1.0	1.5
HPO_4_^2−^	1.0	1.0	1.0	1.0	1.0
SO_4_^2−^	0.5	0.5	0.5	0.5	0.5

**Table 3:** Duration of burn-out test for each scaffold.

Time (hours)	Scaffold composition
1	100 : 0 : 0, 50 : 50 : 0
2	50 : 25 : 25, 0 : 0 : 100, 0 : 50 : 50
3	50 : 0 : 50
